# Intestinal microbiome changes in response to amino acid and micronutrient supplementation: secondary analysis of the AMAZE trial

**DOI:** 10.1017/gmb.2025.10011

**Published:** 2025-09-08

**Authors:** Monica N. Mweetwa, Kazi Ahsan, John Louis-Auguste, Ellen Besa, Joram M. Posma, Nathan P. McNulty, Michael J. Barratt, Jeffrey I. Gordon, Paul Kelly

**Affiliations:** 1 Tropical Gastroenterology & Nutrition group, University of Zambia School of Medicine, Lusaka, Zambia; 2 The Edison Family Center for Genome Sciences and Systems Biology; Center for Gut Microbiome and Nutrition Research, Washington University School of Medicine, St. Louis, USA; 3 Blizard Institute, Queen Mary University of London, London, UK; 4 Department of Metabolism, Digestion and Reproduction, Imperial College, London, UK

**Keywords:** environmental enteropathy, microbiome, small intestine, HIV, nutritional supplementation

## Abstract

Microbial dysbiosis has been linked to environmental enteropathy (EE) and alterations in nutrient absorption; however, compositional modifications following exposure to supplementary nutrients are poorly understood. Here, we report the effect of amino acid and micronutrient supplementation on the gut microbiome of adults with EE.

In the AMAZE trial, adults with EE were randomized to amino acids (AA) and/or micronutrients (MM) for 16 weeks in a 2 × 2 factorial design against placebo. Endoscopy was performed before and after intervention, during which duodenal aspirates were collected as well as fecal samples. 16S rRNA amplicon sequencing was performed on both these samples, and differences in bacterial community composition before and after interventions were investigated using differential abundance analysis, corrected using false discovery rate, plus alpha and beta diversity measurements.

HIV seropositive participants exhibited lower alpha and beta diversity at baseline. AA and/or MM supplementation did not show significant changes in abundance or diversity of genera post-intervention compared to placebo. Micronutrient supplementation resulted in an increase in the pyruvate fermentation to acetone MetaCyc pathways compared to the placebo arm.

This study provides insights into the responsiveness of the gut microbiome to micronutrient and amino acid supplementation in adults with EE.

## Introduction

Originally recognized as “tropical enteropathy,” environmental enteropathy (EE) is widespread in communities that have a high burden of malnutrition and where sanitation is poor (Owino et al., 2016). There is now evidence that it underlies the growth impairment of stunted children globally, leading to delayed neurocognitive development (Wijeakumar et al., 2023), blunted responses to oral vaccines (Bhattacharjee et al., 2021), perturbations in the gut microbiome (Bartelt et al., 2019; (Cowardin et al., 2023; Gizaw et al., 2022; Chaima et al., 2021), and impaired absorption of nutrients (Morais and Silva, 2019). Micronutrient and amino acid deficiencies exacerbate these adverse effects, as they are crucial for immune function, cellular repair, and overall growth (Guerrant et al., 2016; Zyambo et al., 2022; McCormick et al., 2019; Manary et al., 2010).

Recent research has illuminated the critical role of the gut microbiome in the context of EE (Chen et al., 2020). The gut microbiome, comprising trillions of microorganisms, is integral to maintaining intestinal health, aiding in digestion, synthesizing a range of essential nutrients, and modulating the immune system (Gomaa, 2020). In individuals with EE, epithelial barrier function and villus height are reduced with associated alterations in the composition of the gut microbial community, inflammation, and impaired nutrient absorption (Cowardin et al., 2023). Dysbiosis, a broad term describing an imbalance in the microbial community, can exacerbate the malabsorption of micronutrients and amino acids, further hindering growth and development. However, dysbiosis is often ill-defined, especially in the small intestine, which is the focus of only a small number of studies. There is emerging evidence that supplementation with amino acids or micronutrients such as zinc and B vitamins (Guetterman et al., 2022; Zhang et al., 2023) can drive a change in the gut microbiome to a more stable and host beneficial community. However, this has not been explored in the context of EE to date.

We previously demonstrated that amino acid supplementation increased small intestinal villus height and mitigated barrier impairment when combined with micronutrients in adults with EE (Louis-Auguste et al., 2019). Furthermore, there was an increase in co-metabolites such as β-hydroxy-β-methyl butyrate in these adults following amino acid supplementation. Building upon this groundwork, our study aimed to investigate the impact of micronutrient and/or amino acid supplementation on the proximal small intestinal and fecal microbial communities including alpha and beta diversity metrics in Zambian adults with EE. We also sought to understand the relationship between the duodenal and fecal microbiome with sample characteristics such as HIV status at baseline in these individuals.

## Methods

### AMAZE Study

Adults deemed likely to have EE (based on histological examination of duodenal biopsies) were recruited from Misisi, Lusaka, where we have previously conducted studies on EE (Kelly et al., 2004, 2008, 2016). They were invited to take part in the AMAZE study, whose design has been described previously (Louis-Auguste et al., 2019). In brief, 102 adults were recruited between October 2015 and May 2016 and randomized into amino acid (tryptophan, leucine, and glutamine) and/or micronutrient supplementation arms for 16 weeks in a 2 × 2 factorial comparison against placebo. Composition of the supplements and placebos is provided in Tables 1 and 2. Endoscopy was done before and after the intervention. This clinical trial was approved by the University of Zambia Biomedical Research Ethics Committee (ref 007-11–14, dated 22 January, 2015), the Zambia Medicines Regulatory Authority (reference CT054/15), and the National Health Research Authority of Zambia (29 October, 2015), and registered with the Pan African Clinical Trials Registry (PACTR201505001104412).

**Table 1.** Composition of the amino acid supplement

	Active	Placebo
l-Gln	29.9 g	
l-Trp	0.28 g	
l-Leu	2.79 g	
Maltodextrin		32.9 g
Sodium hydrogen sulphite (E222)		0.07 g
Total	33 g	33 g

**Table 2.** Composition of the micronutrient supplementation

	Average dose per tablet	Total daily dose	EC RDA	Multiples of EC RDA per daily dose
Vitamin A RE (IU/μg)	2664/800	5328/1600	800	2
Vitamin D3 (IU/μg)	400/10	800/20	5	4
Vitamin E α-TE (mg)	40	80	12	6.66
Vitamin K (μg)	70	140	75	1.86
Vitamin C (mg)	150	300	80	3.76
Thiamin (mg)	18	36	1.1	32.72
Riboflavin (mg)	6	12	1.4	8.58
Niacin NE (mg)	27	54	16	3.38
Vitamin B6 (mg)	10	20	1.4	14.28
Folic acid (μg)	400	800	200	4
Vitamin B12 (μg)	14	28	2.5	11.2
Pantothenic acid (mg)	20	40	6	6.66
Iron (mg)	8	16	14	1.14
Magnesium (mg)	60	120	375	0.32
Zinc (mg)	15	30	10	3
Iodine (μg)	200	400	150	2.66
Copper (mg)	500	1000	1	1
Manganese (mg)	4	8	2	4
Selenium (μg)	180	360	55	6.54
Chromium (μg)	100	200	40	5
L-Cystine (mg)	40	80	N/A	N/A
L-Carnitine (mg)	30	60	N/A	N/A
Citrus Bioflavonoids (mg)	30	60	N/A	N/A
Betacarotene (Natural Carotenoids) (mg)	3	6	N/A	N/A

Excipients: Microcrystalline cellulose; hydroxypropylmethylcellulose; ethyl cellulose; propylene glycol; purified talc; titanium dioxide; iron oxides; glucose syrup; purified talc; magnesium stearate; silicon dioxide; polyvinylpolypyrrolidone; acacia; sucrose; starch; tricalcium phosphate; dicalcium phosphate; medium chain triglycerides; colloidal silica; maltodextrin; butylated hydroxyanisole.

EC RDA, European Community Recommended Daily Allowance; RE, retinol equivalents; IU, international units; α-TE, alpha-tocopherol equivalents; NE, niacin equivalents. Taken from (European Union, 2008). The micronutrient placebo contained only the excipients listed above.

### Sample collection

Fecal samples were collected before endoscopy at baseline and after 16 weeks of intervention. Duodenal aspirates were collected during endoscopy, before and after the intervention period. These samples were stored at −80°C before transportation to Washington University, St. Louis for V4 16S rRNA amplicon sequencing. Blood samples were also collected before endoscopy at both timepoints for enteropathy marker testing.

### Histomorphometric analysis

Morphometry was carried out on well-orientated fixed tissue as previously described (Louis-Auguste et al., 2019; Mulenga et al., 2022). In brief, 3 μm thick H&E-stained sections were scanned on an Olympus VS-120 microscope. Sections where crypts could be seen to have been sectioned longitudinally were considered suitable, and the villus-crypt boundary was defined visually. Crypt depth was measured from the crypt base to the boundary, villi from the boundary to the villus tip, and villus width perpendicular to villus height.

### Biomarkers of enteropathy

The presence of Lipopolysaccharide (LPS) was analyzed using the Pyrochrome LAL endpoint assay (Associates of Cape Cod International Inc, East Falmouth, MA, USA). Soluble CD14 (sCD14) and C-reactive protein (CRP) were analyzed by ELISA (R&D systems, Minneapolis, MN, USA) as biomarkers of microbial translocation and systemic inflammation, respectively. All assays were run as per manufacturer’s instructions using blood samples.

### DNA extraction and sequencing

#### Duodenal aspirates

Parent stocks of frozen duodenal aspirates were thawed, and a 100 μL aliquot of each was prepared in an anaerobic Coy chamber. *Alicyclobacillus acidiphilus* DSM 14558 (9.90 × 10^5^ cells/sample) was added as a spike-in reference control. The aspirate samples were centrifuged at 5000 × *g* for 10 min at 10°C in 2 mL Axygen screw cap tubes. After removing the supernatant, the pellet was enzyme digested by adding 50 μL of 2 μg/μL proteinase K (PicoPure DNA Extraction Kit, ThermoFisher) for 10 h at 65°C followed by a 10 min incubation at 95°C to inactivate the enzyme. A phenol–chloroform bead beating DNA extraction protocol was followed by adding 250 μL 0.1 mm zirconia silica beads, one 3.97 mm steel ball, 710 μL of a 500:210 mixture of 2X buffer A (0.2 M NaCl, 0.2 M Tris, 0.02 M EDTA) and 20% SDS, and 500 μL of pH 8 phenol:chloroform:isoamyl alcohol. Bead beating was performed on a Biospec Minibeadbeater-96 for 4 min. DNA was purified using Qiaquick columns (Qiagen), eluted in 70 μL Tris-EDTA (TE) buffer, quantified (Quant-iT dsDNA broad range kit; Invitrogen) and normalized to 2 ng/μL. Hypervariable region 4 (V4) of the bacterial 16S rRNA gene was amplified using barcoded 515F and 806R primers, and amplicon libraries were sequenced (2 × 250 nt paired-end reads; Illumina MiSeq) to a depth of 7.73 × 10^4^ ± 1.51 × 10^5^ (mean ± SD) reads/duodenal sample.

#### Feces

Frozen fecal samples were pulverized in liquid nitrogen using a mortar and pestle inside a biosafety cabinet, and an approximately 200 mg aliquot of each sample was prepared. Each aliquot was subjected to bead beating with 500 μL of 0.1 mm diameter zirconia/silica beads in 500 μL phenol: chloroform: isoamyl alcohol (25:24:1), 210 μL 20% SDS, and 500 μL buffer A (0.2 M NaCl, 0.2 M Tris, 0.2 M EDTA) for 4 minutes (Biospec Minibeadbeater-96). DNA was subsequently purified, quantified, normalized, and sequenced as described for the duodenal aspirate samples, to a depth of 6.19 × 10^4^ ± 6.66 × 10^3^ sequence reads/sample.

### Bioinformatic and statistical analysis

Statistical analyses and visualizations were conducted in R v4.4.1 using ape.v5.8 (Paradis and Schliep, 2019), DADA2.v1.3 (Callahan et al., 2016), ggplot2.v3.5.1 (Wickham, 2016), Masslin2.v1.16 (Mallick et al., 2021), MicrobiomStat.v1.2 (Yang, 2024), MicroViz.v0.12.1 (Barnett et al., 2021), msa.v1.34 (Bonatesta, 2017), Phyloseq.v1.46 (McMurdie and Holmes, 2013), and vegan.v2.6–6.1 (Oksanen et al., 2022) R packages.

***Descriptive statistics*** of the sample features were compared using chi-squared test for categorical variables and Kruskal Wallis test for continuous variables. Comparisons of change in biomarkers and morphometry measures between groups was done using a 2-way ANOVA test using the ez.v4.4.0 r package (Lawrence, 2016).

***ASV identification and quantitation***: Paired-end 250 nucleotide reads were demultiplexed, and a DADA2-based custom pipeline (see Data Availability) was used to denoise and identify amplicon sequence variants (ASVs). Taxonomic assignment was performed in R 4.3.1 using a Naïve Bayes Classifier included with DADA2 based on the SILVA rRNA database (release 138.1). To determine the bacterial load within each duodenal aspirate sample (i) raw ASV counts were transformed to relative abundances, (ii) the relative abundances of any ASVs identified as spike-in-derived (based on an *Alicyclobacillus* genus assignment) were summed, and (iii) bacterial load [cells per mL of sample] was calculated as (# spike-in cells added per mL sample) × ((1 – spike-in relative abundance)/spike-in relative abundance). Spike-in organisms were then removed from the count abundance table, and raw counts were normalized using size factors estimated by DESeq2 to account for differences in library size. Relative abundances were than calculated from these normalized counts and scaled by the bacterial load for the respective sample to calculate absolute abundances. ASVs with ambiguous taxonomic assignment at species level were filtered out leaving 1,245 ASVs from 138 duodenal samples and 3,271 ASVs from 158 fecal samples. Multiple sequence alignment was performed using msa.v1.34 package (Bonatesta, 2017) in R with default settings on ASV sequences then used as input to create a distance matrix for phylogenetic tree construction using the neighbor joining algorithm. The ASVs were then merged to create a dataset with 4,350 ASVs mapping to 399 genera. All downstream analysis was carried out on absolute abundance estimates at genus level expect for alpha diversity analysis, which was done at species level.

***Diversity analysis***: alpha-diversity was quantified using Faith’s phylogenetic diversity (PD) metric on the phylogenetic distance between species, while beta diversity was quantified using Bray–Curtis distances on absolute abundance estimates. The relationship between alpha diversity and sample characteristics at baseline was modelled using linear regression models. A 2-way ANOVA test was used to compare alpha diversity within timepoints, between intervention arm and the interaction of the two with subject treated as a random effect, while a nonparametric multivariate analysis of variance (PERMANOVA) was performed for beta diversity.

***Baseline microbiome associations***: Multivariable linear regression of was used to assess the associations between the total sum scaled (TSS) abundance of individual genera present in at least 10% of samples with histomorphometry measures and enteropathy biomarkers, adjusting for HIV status and age. Zero values were replaced with half the minimum value prior to log transformation. All *p*-values were corrected for multiple testing using the Benjamini–Yekutieli false discovery rate with pFDR ≤0.05.

***Pathway prediction***: Potential pathway predictions were generated using Phylogenetic Investigation of Communities by Reconstruction of unobserved states (PICRUSt2) (Douglas et al., 2020) on ASVs absolute counts. PICRUSt2 utilizes 16S sequencing data to predict KO metagenomes and EC numbers, which can then be used to infer MetaCyc pathways present in samples using MinPath.

***Differential abundance***:

Differential abundance of individual genera between HIV status at baseline, sample type at baseline, and within group post-intervention changes were assessed using negative-binomial generalized regression on trimmed mean (TMM) normalized counts using the Masslin2.v1.16 (Mallick et al., 2021) r package. Differences in predicted MetaCyc pathways between sample types was modelled using negative-binomial generalized regression on trimmed mean (TMM) normalized counts using the MicrobiomeStat R package.

Changes in genera and MetaCyc pathway abundance post-intervention in nutritional arms compared to the placebo arm were assessed using log-linear models on TSS normalized counts with bias correction by winorization at the 95th percentile to reduce the influence of outliers using the MicrobiomeStat R package.

All *p*-values were corrected for multiple testing using the Benjamini–Yekutieli False Discovery Rate with pFDR ≤0.05 indicating significance.

Code and data used in this analysis is available on GitLab at https://gitlab.com/Gordon_Lab/amplicon_sequencing_pipeline and GitHub at https://github.com/Monica-Mweetwa/AMAZEMicrobiomeAnalysis.git

## Results

### Sub-study population characteristics and phenotypes

The AMAZE trial recruited 102 participants. Paired (pre- and post-intervention) duodenal or fecal microbiome data were available for 83 of these participants. While fecal samples were available from 79 participants, only 69 duodenal aspirates were available, and both samples were available from 64 participants (Supplementary Figure S1). Table 3 shows the demographic, biomarker, and morphometry data for this cohort which, as previously reported (Louis-Auguste et al., 2019), was seasonally biased. As this analysis draws on a subset of 83 of the original 102 trial participants, statistical hypothesis testing was conducted to compare groups, but there were no differences in participant characteristics between intervention arms.

**Table 3.** Demographic and nutritional characteristics of participants in each intervention arm

	Amino acid (*N* = 22)		Amino acid and Micronutrient (*N* = 20)		Micronutrient (*N* = 21)		Placebo (*N* = 20)		*p*
	Pre	Post	Pre	Post	Pre	Post	Pre	Post	
Age at baseline (years)	35.5 [18.0, 60.0]		32.0 [21.0, 54.0]		43.0 [21.0, 57.0]		38.5 [19.0, 57.0]		0.303
Sex									
Female	11		15		15		16		0.157
Male	11		5		6		4		
HIV status									
Negative	16		12		12		16		0.407
Positive	6		7		9		4		
BMI, kg/m^2^	21.0 [19.0, 31.0]	22.0 [18.0, 32.0]	23.0 [18.0, 51.0]	23.0 [17.0, 50.0]	22.5 [19.0, 39.0]	23.0 [18.0, 38.0]	22.0 [19.0, 37.0]	22.0 [19.0, 36.0]	0.461^a^
MUAC, cm	27.5 [24.0, 37.0]	29.0 [22.0, 34.0]	27.5 [24.0, 43.0]	27.0 [22.0, 42.0]	30.0 [23.0, 40.0]	30.5 [23.0, 37.0]	27.5 [23.0, 38.0]	27.0 [22.0, 39.0]	0.702^a^
LPS, EU/mL	138 [0, 604]	161 [11.0, 445]	79.0 [0, 689]	187 [11.0, 524]	117 [0, 384]	165 [50.0, 490]	110 [0, 653]	139 [20.0, 415]	0.353^a^
CRP, ng/mL	1950 [207, 52500]	1890 [81.0, 29500]	2400 [106, 52500]	1520 [135, 25100]	3600 [236, 52500]	2840 [47.0, 18700]	1700 [110, 18900]	5470 [299, 19000]	0.369 ^a^
sCD14, ng/mL	1890 [1140, 5020]	1860 [1080, 3590]	2190 [319, 14700]	1770 [1110, 4210]	2210 [984, 6760]	2160 [1260, 5780]	1740 [1110, 5640]	1680 [914, 3180]	0.182^a^
Crypt depth, μm	185 [122, 339]	153 [99.0, 197]	163 [109, 215]	149 [107, 197]	156 [105, 241]	128 [90.0, 169]	168 [120, 211]	134 [110, 224]	0.331^a^
Villus height, μm	176 [101, 262]	197 [130, 262]	197 [107, 472]	196 [133, 261]	209 [160, 307]	196 [103, 279]	226 [159, 292]	189 [168, 366]	0.263^a^
Villus width, μm	236 [138, 378]	226 [143, 455]	217 [179, 369]	230 [155, 275]	231 [196, 424]	227 [142, 341]	210 [131, 498]	243 [124, 630]	0.310^a^

*Note:* All continuous values are given as median [Min Max].

Abbreviations: MUAC = middle upper arm circumference; Pre = pre-intervention; Post = post-intervention.

a These *p*-values represent the interaction between timepoint and intervention arm. Output for intervention arm and timepoint separately are shown in Supplementary Table S1.

### Baseline microbial characteristics

**Figure 1.**
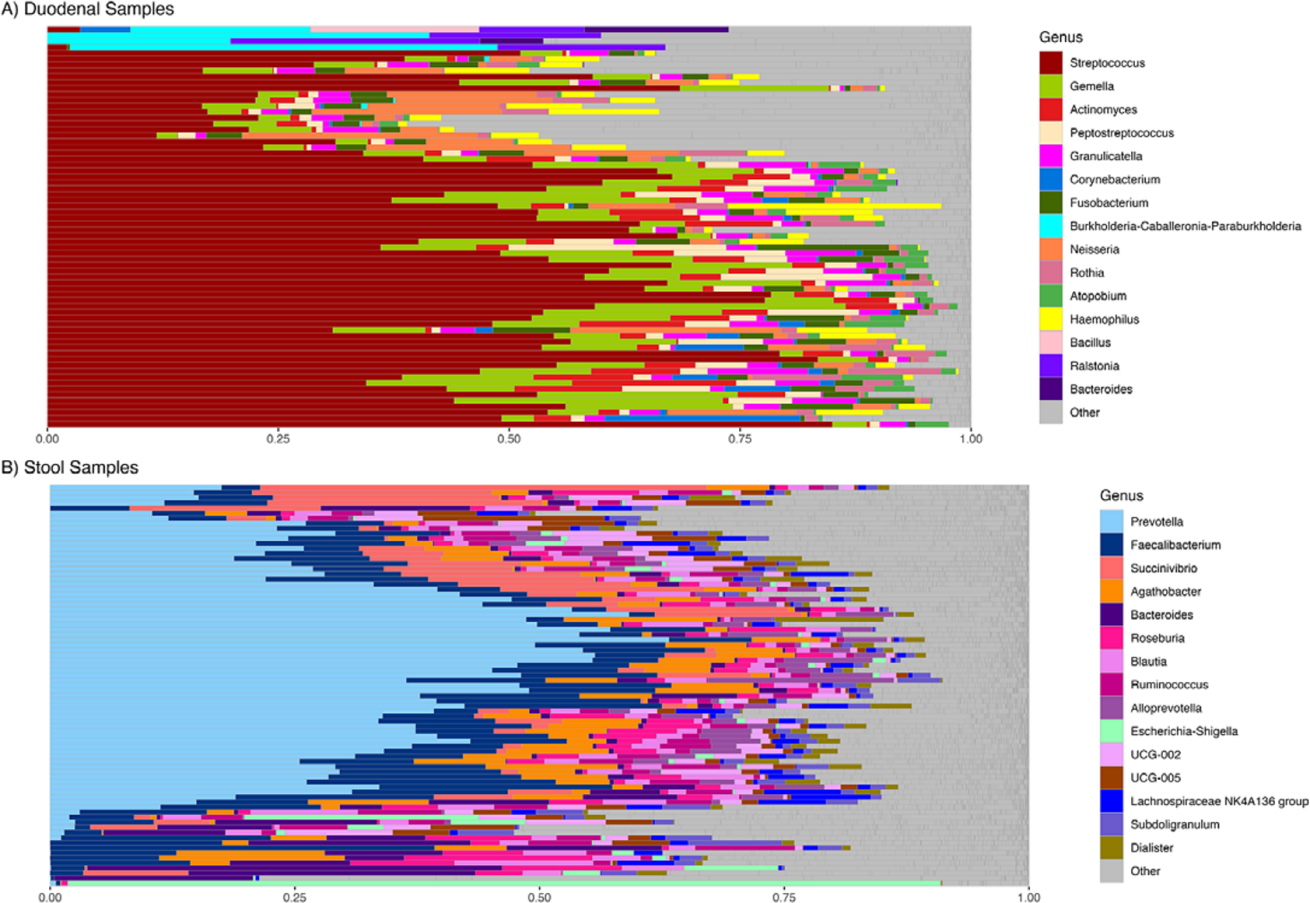
Relative abundance of genera at baseline. (A) In duodenal sample and (B) in fecal (stool) samples.

At baseline, the duodenal aspirate communities of study participants contained members of the phyla Firmicutes, Proteobacteria, Bacteroidota and Actinobacteriota, represented primarily by members of the genera *Streptococcus*, *Gemella*, *Actinomyces*, *Peptostreptococcus*, and *Granulicatella* (Figure 1A, Supplementary Figure S2A) with *Streptococcus* comprising 45.8 ± 20.5% (mean ± SD) of all reads. Fecal communities contained the phyla Firmicutes, Proteobacteria, and Bacteroidota, represented primarily by *Prevotella*, *Faecalibacterium*, *Succinivibrio*, *Agathobacter*, *Bacteroides*, *Roseburia*, and *Blautia*, with *Prevotella* being most abundant (27.9 ± 18.3% of all reads) (Figure 1B, Supplementary Figure S2B).

Duodenal alpha diversity was not different when comparing HIV positive and negative individuals, but HIV positive individuals had significantly lower fecal alpha diversity at baseline measured by Faith’s phylogenetic diversity (PD) richness (Supplementary Table S2). In terms of beta diversity, age and sCD14 concentration in serum were positively associated with duodenal diversity (*R*^2^ = 0.052, *p* = 0.006; *R*^2^ = 0.038, *p* = 0.025 respectively), whereas HIV status accounted for the greatest community variation in feces (Supplementary Table S3); the latter was driven mainly by the presence of more *Anaerosporobacter*, *dgA.11.gut.group, Pseudocitrobacter*, *Megasphaera*, *Tyzzerella*, and *Megamonas* and reduced detection of *Escherichia/Shigella*, *Lachnospiraceae*, and *Alistipes* in HIV positive stool (Supplementary Figure S3). Neither alpha nor beta diversity correlated with any histomorphometric measures (Supplementary Tables S2 and S3).

The abundance of *Capnocytophaga and Bergeyella* in the duodenum was significantly positively associated with CRP concentrations in serum (Supplementary Figure S4A) while *Intestinibacter* and *Flavonifractor* abundance in fecal was negatively associated with CRP concentrations (Supplementary Figure S4B).

**Figure 2.**
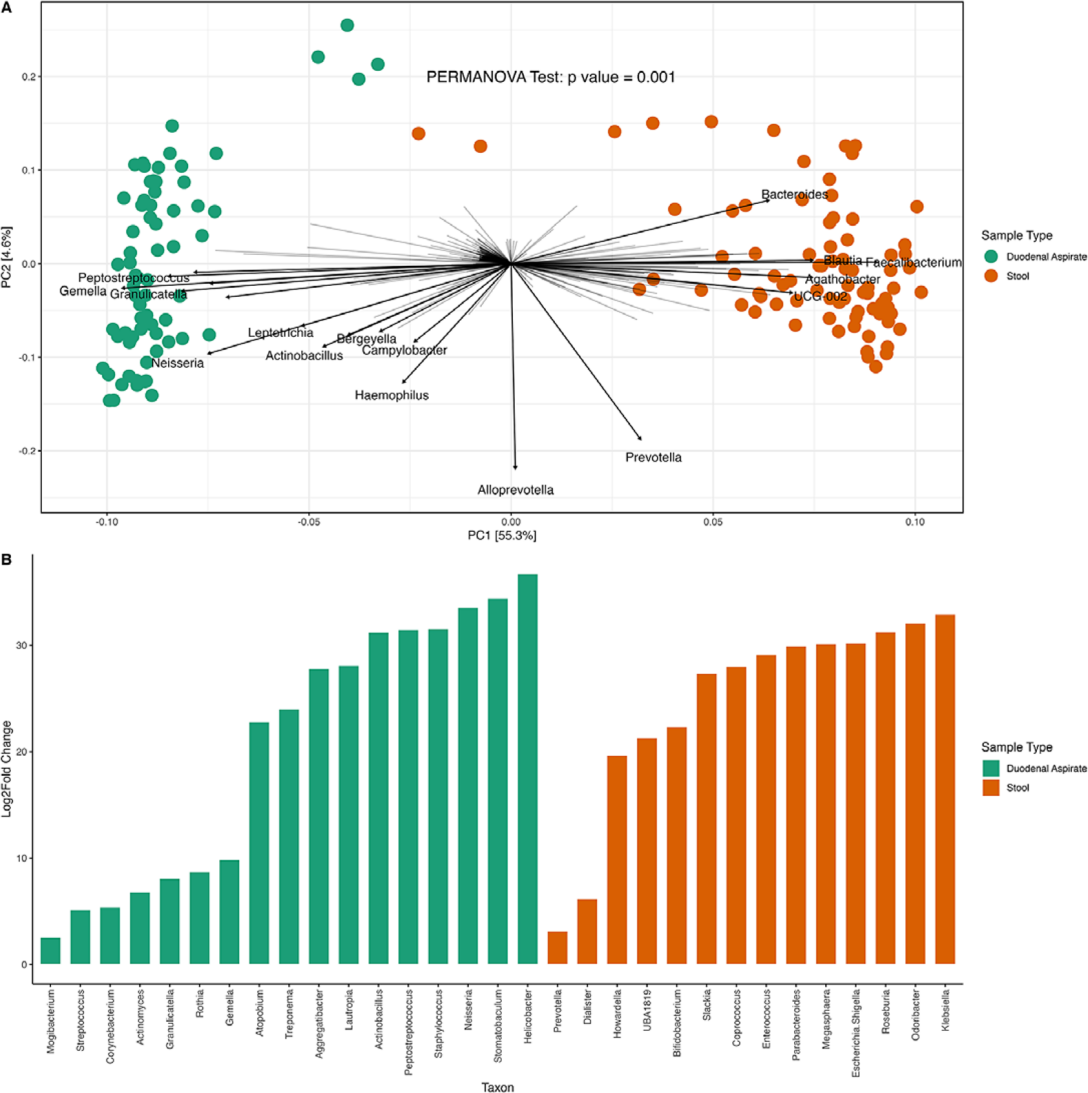
Microbial differences between duodenal and fecal (stool) samples. (A) PCA plot depicting sample similarities, colored by sample type. (B) Genera associated with sample type.

PCA on centered log ratio transformed absolute counts disclosed distinct clustering between duodenal and fecal communities (Figure 2A) driven by the presence of *Streptococcus*, *Gemella*, *Neisseria*, *Granulicatella*, and *Peptostreptococcus* in duodenal samples and *Faecalibacterium*, *Prevotella*, and *Ruminococcus* in feces (Figure 2B).

We assessed the differences in predicted MetaCyc pathways abundances in each sample type using linear regression models and observed that the pathways most strongly associated with the duodenal microbiome were preQ0 biosynthesis (PWY-6703) (Supplementary Figure S5) and L-methionine biosynthesis (PWY-5345) for the fecal microbiome (Supplementary Figure S5).

### The effect of supplementation on community composition and potential function

**Figure 3.**
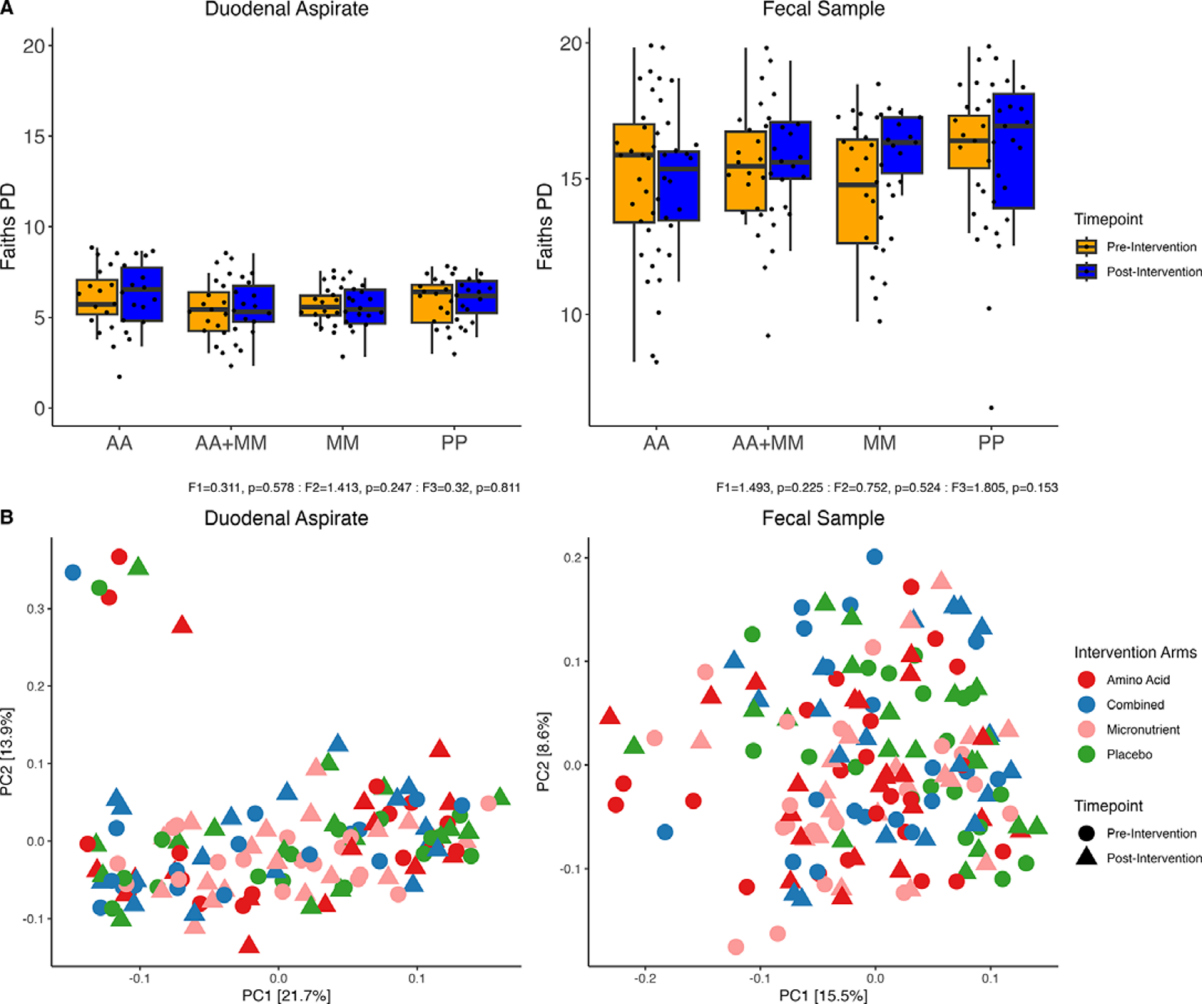
Microbial changes post intervention. A) Alpha diversity pre- and post- intervention in duodenal and fecal samples where F1 is the F-Score for timepoint, F2 for intervention arm and F3 for the interaction between timepoint and intervention arm. B) PCA plots of Bray–Curtis distances colored by intervention arm and shaped by study timepoint.

Post-intervention analysis revealed no significant changes in overall microbial richness in both duodenal and fecal samples, either within intervention (before/after) or between treatment arms (Figure 3A). Community diversity, as measured by Bray–Curtis distances, also showed no significant changes following either intervention (Figure 3B). Similar results were observed when comparing amino acid intervention to no amino acid intervention.

Changes in genus abundance post-intervention highlighted some noteworthy shifts for each nutritional intervention (Table 4). In duodenal aspirates, there was a significant decrease in *Arcanobacterium*, *Lawsonella*, *Mycoplasma*, and *Tropheryma* abundance following amino acid intervention. There was an increase in *Olsenella* and *Peptoniphilus* within the combined arm. Supplementation with only micronutrients resulted in a significant decrease in *Lactobacillus* and *Lauptria* abundance and an increase in *Bifidobacterium. Bifidobacterium* is depleted in children with impaired intestinal barrier function and zinc deficiency (Chai et al., 2024) and has been shown to respond positively to elemental iron in culture (Ostrov et al., 2024) and folic acid (Zheng et al., 2024). These changes did not remain significant when compared to the placebo group (Supplementary Figure S6). Distribution of the absolute abundances for these is shown in Supplementary Figure S7, which shows that these changes were largely driven by samples with very high or very low abundance. However, some data may be still meaningful for example, *Lauptria* in the micronutrient arm showed a significant decrease in abundance even after manual inspection.

**Table 4.** Significant differences in abundances of genera post-intervention within each intervention arm using negative-binomial regression models

Sample type	Intervention arm	Genus	β	SE	pFDR
Duodenal aspirate	Amino acid	*Arcanobacterium*	−4.071	0.001	0E+00
		*Lawsonella*	−17.451	4.971	3.4E−02
		*Mycoplasma*	−17.162	3.336	3.41E−05
		*Tropheryma*	−1.673	0.001	0E+00
	Combined intervention	*Olsenella*	0.059	0.002	2.51E−190
		*Peptoniphilus*	0.815	0.003	0E+00
	Micronutrient	*Bifidobacterium*	16.386	3.203	3.05E−05
		*Lactobacillus*	−16.661	4.486	1.49E−02
		*Lautropia*	−23.952	3.766	5.86E−08
		*Methylobacterium-Methylorubrum*	−18.773	3.139	3.23E−07
	Placebo	*Dolosigranulum*	−17.278	3.993	1.19E−03
		*Enterococcus*	0.832	0.0001	0E+00
		*Mobiluncus*	−14.62	3.492	1.78E−03
		*Murdochiella*	15.484	4.186	1.14E−02
Fecal Sample	Combined Intervention	*Acidaminococcus*	−3.131	0.471	9.70E−09
		*Cetobacterium*	0.948	0.094	2.22E−21
		*Ruminobacter*	−2.655	0.049	0E+00
	Micronutrient	*Campylobacter*	0.522	0.107	2.5E−04
		*Cellulosilyticum*	2.969	0.102	2.61E−183
		*Hungatella*	−2.826	0.340	3.41E−14
	Placebo	*Megamonas*	−2.759	0.328	3.63E−14
		*Pyramidobacter*	−1.633	0.276	2.36E−13
		*Raoultella*	−1.174	0.242	4.22E−04

*Note:* The β coefficient represents the change in the log of the expected count post-intervention compared to pre-intervention.

There was a decrease in *Dolosigranulum* and *Murdochiella* post placebo. Several gut bacteria have been reported to metabolize maltodextrin, the dominant compound in the placebo treatment, so this may contribute to its lack of “inertness” in both duodenal and fecal samples.

In fecal samples, combined intervention of amino acids and micronutrients resulted in an increase in *Cetobacterium* and a decrease in *Acidaminococcus* and *Enterococcus* post-intervention. Supplementation with micronutrients alone increased abundance of *Campylobacter*, *Cellulosilyticum* and decreased abundance of *Hungatella* in feces (Table 4). When compared to the placebo arm, none of these remained significant (Supplementary Figure 7). Distribution of the absolute abundances for these are shown in Supplementary Figure 8.

To assess the possible metabolic impact of the modest changes observed in composition, we analyzed their predicted functional consequences. Differential abundance of MetaCyc pathways following intervention showed an increase in the pyruvate fermentation to acetone (MetaCyc ID: PWY-6588) in fecal samples in the micronutrient arm compared to placebo (β = 1.28, pFDR = 0.043, Prevalence = 90.5%, Mean abundance = 0.0015). There were no other significant changes in potential pathways post-intervention compared to the placebo arm (Supplementary Figure S9).

## Discussion

We explored the effects of amino acid and/or micronutrient interventions on the gut microbiome in Zambian adults with EE by looking at their diversity metrics, changes in composition and predicted pathway associations in the bacterial communities present in duodenal aspirates and fecal samples obtained from study participants. The duodenal microbial community was dominated by *Streptococcus*, *Gemella, Actinomyces, Peptostreptococcus*, and *Granulicatella*, whereas the fecal community was primarily composed of *Prevotella, Faecalibacterium, Succinivibrio*, *Agathobacter*, *Bacteroides, Roseburi*a, and *Blautia.* This compartmentalization underscores the importance of considering the unique microbial environments along the GI tract when designing and interpreting gut microbiome studies. Although upper and lower gut bacterial communities are clearly distinct, there are interesting correlations which might reflect host molecular profiles, or interactions between microbial communities at distant anatomical sites. The presence of microbes first recognized as oral commensals, such as *Neisseria*, *Gemella*, *Granulicatella*, and *Peptostreptococcus* in duodenal samples is noteworthy, as these genera have been associated with EE and stunting in children from East Asian and Southern African populations (Chen et al., 2020; Douglas et al., 2020; Donowitz et al., 2016; Monira et al., 2011; Vonaesch et al., 2018). These findings suggest that the establishment of members of the oral cavity in the duodenum may be a general feature of EE; a similar observation was made in stunted children from CAR and Madagascar (Vonaesch et al., 2018).

The reduced fecal alpha diversity in HIV-seropositive individuals with EE is consistent with other studies in African adults with HIV (Parbie et al., 2021; Rocafort et al., 2019; Shenoy et al., 2019). In Zambia, these individuals had higher *Pseudocitrobacter, Megasphaera, Tyzzerella*, and *Megamonas*, and decreased levels of *Escherichia/Shigella*, *Lachnospiraceae*, and *Alistipes* compared to their HIV-negative counterparts, whereas in HIV-positive individuals in Uganda, members of the families *Prevotellaceae* and *Streptococcaceae* dominated (Shenoy et al., 2019). Key taxa detected in Ghanaian individuals were *Dorea* and *Blautia* (Parbie et al., 2021). A common feature seen in sub-Saharan studies is the reduced detection of *E. coli* and *Shigella*, possibly owing to frequent antibiotic exposure. In the national referral hospital in Zambia, high levels of multidrug-resistant *E. coli* have been documented in HIV-positive individuals (Chabala et al., 2020) which supports the idea that frequent antibiotic exposure has important effects on the gut microbiome.

### Effects of supplementation on microbial composition

Post-intervention analyses indicated that neither micronutrient nor amino acid supplementation significantly altered the overall alpha or beta diversity of the microbiome in both duodenal and fecal samples. Amino acid supplementation was associated with a decrease in the abundance of *Mycoplasma* spp. in duodenal samples. Increased abundance of *Mycoplasma* spp. in the intestines has been associated with inflammatory bowel disease (Chen et al., 2001), suggesting amino acids might protect against *Mycoplasma* colonization in the small bowel.

Both *Lactobacillus* and *Bifidobacterium* are commonly used probiotics that have been shown to increase in abundance after micronutrient supplementation (Mikulic et al., 2024; Pavel et al., 2023; Li et al., 2024; Schalich et al., 2024). Our analysis showed that *Bifidobacterium* increased in abundance and *Lactobacillus* reduced in abundance in duodenal samples after micronutrient intervention alone. Iron supplementation in children has shown an increase in *Lactobacillus* and a decrease in *Bifidobacterium* (Sjödin et al., 2019) contrary to our findings. Species in these genera have similar properties (i.e., they are typically non-spore-forming, gram-positive, and produce lactic acid). One difference between them is that bifidobacteria are strict anaerobes that produce SCFA, whereas *Lactobacillus* spp. are facultative anaerobes that predominantly refine sugars to produce lactic acid. Together with the decrease in *Bifidobacterium* abundance in feces, these observations might signify a shift in oxygen concentrations along the GI tract post micronutrient supplementation. Measures of pH and oxygen concentration in future studies are needed to explore this hypothesis further.

The combined intervention increased the representation of taxa known to metabolize and/or utilize micronutrients and amino acids including *Peptoniphilus* and *Olsenella.* These genera both possess enzymes that are capable of utilizing glutamine (glutaminase, EC 3.5.1.2) and leucine (leucine dehydrogenase, EC 1.4.1.9) (Chang et al., 2015). Furthermore, these enzymes would initiate the deamination of glutamate, lysine, glycine, and aspartate, which would then result in the production of short-chain fatty acids (SCFAs) (Beaumont et al., 2022). Deamination of amino acids also releases ammonia, which can negatively impair mitochondrial respiration, causing decreased cell proliferation in the intestinal epithelium (Beaumont et al., 2022). The *Peptoniphilus* genus includes butyrate-producing, non-saccharolytic species that utilize amino acids as a major energy source (Ashniev et al., 2022). *Olsenella* has been detected in adults with altered vitamin D levels (Nitzan et al., 2023) and has been shown to respond to iron and zinc supplementation in animal models (Ishaq et al., 2019).

In fecal samples, the combined intervention was associated with an increase in *Cetobacterium* abundance and a decrease in *Acidaminococcus* and *Enterococcus.* These changes also have the potential to modify gut health because all three of these genera contain species that possess an enzymatic capacity to utilize micronutrients and amino acids (Qian et al., 2024; Veith et al., 2015). Micronutrient supplementation alone was associated with increased abundance of *Campylobacter* and *Cellulosilyticum*, and reduced abundance of *Hungatella. Campylobacter* spp. can readily utilize a variety of amino acids and micronutrients in the gut (Indikova et al., 2015; Stahl et al., 2012), particularly *Campylobacter jejuni.* Representation of the pathway for pyruvate fermentation to acetone was also significantly elevated following micronutrient treatment, suggesting enhanced short-chain fatty acid production, which is beneficial for gut health (Xiong et al., 2022). We previously showed (Louis-Auguste et al., 2019) through LCMS that compared to placebo, Micronutrients resulted in elevated Pantothenate, HMB, succinate, 2-PY and NMND. The fermentation of some of these metabolites such as succinate is a part of the pyruvate fermentation pathways. Therefore, fermentation capacity seems to be influenced by micronutrients. Succinate is also sensed by the gut as one pathway of recognition of the presence of the microbiota.

## Conclusions

The study explored the potential differential effects of micronutrient and amino acid supplementation on the gut microbiome in adults with EE. While overall microbial richness and diversity remained unchanged, some shifts in genus abundance and functional pathways were observed within intervention arms. However, these changes were not significantly different from the placebo group, highlighting the need for future studies to determine whether such microbial changes are physiologically meaningful and beneficial.

## Limitations

- This analysis was constrained by the small sample size, which was a subsidiary analysis of a larger study.
- There is a possibility of contamination of upper GI microbes in duodenal fluid from the endoscope during sample collection. Future work should try to minimize this by using sampling techniques that reduce the interaction of fluid along the GI tract such as aspiration tubes.
- Our analyses were based on 16S rRNA amplicon datasets (ASVs) that are limited in their ability to resolve species and strain level variation. Future work should include deep sequencing of shotgun metagenomic/transcriptomic datasets and assembly/annotation of metagenome assembled genomes (MAGs) to more precisely define the functional output of the microbiomes as a function of biogeography and treatment.
- The inherent sparsity in the data is a potential source of limitation in our findings for the differential abundance analysis over time for each intervention arm.

## Supporting information

Mweetwa et al. supplementary material Mweetwa et al. supplementary material

## Data Availability

The 16S RNA dataset presented will be submitted to SRA archive under accession number PRJNA1153957 The code used for processing of the raw data is available on GitLab at https://gitlab.com/Gordon_Lab/amplicon_sequencing_pipeline The code and data used for statistical analysis and generation of figures in this manuscript are available on GitHub at https://github.com/Monica-Mweetwa/AMAZEMicrobiomeAnalysis.git

## Supplementary material

The supplementary material for this article can be found at http://doi.org/10.1017/gmb.2025.10011.
