# Intestinal microbiome changes in response to amino acid and micronutrient supplementation: secondary analysis of the AMAZE trial – CORRIGENDUM

**DOI:** 10.1017/gmb.2026.10025

**Published:** 2026-05-07

**Authors:** Monica N. Mweetwa, Kazi Ahsan, John Louis-Auguste, Ellen Besa, Joram M. Posma, Nathan P. McNulty, Michael J. Barratt, Jeffrey I. Gordon, Paul Kelly

The original article was published with errors in three author names. The names were incorrectly listed as Nathan McNulty, Micheal J. Barrat, and Jeff Gordon. The correct names are Nathan P. McNulty, Michael J. Barratt, and Jeffrey I. Gordon. The authors apologise for this error, which has now been corrected in both the PDF and HTML versions of the article.

