# Supplementary material for: Intestinal microbiome changes in response to amino acid and micronutrient supplementation: secondary analysis of the AMAZE trial
Source: Gut Microbiome (Camb). 2025 Sep 8;6:e16. doi: 10.1017/gmb.2025.10011 (PMC12509164; doi:10.1017/gmb.2025.10011)
Supplement: Mweetwa et al. supplementary material [file S263228972510011Xsup001.pdf]

Intestinal microbiome changes in response to amino acid and micronutrient supplementation: secondary analysis of the AMAZE Trial

Mweetwa et al

Supplementary Tables and Figures

Supplementary Table 1: Two-way ANOVA analysis of enteropathy markers and morphometry measures within timepoints and between intervention arms.

		Timepoint			Intervention arm			Interaction (Timepoint : Intervention arm)		
	N	df	F1	p	df	F2	p	df	F3	p
**BMI, kg/m²**	74	**1**	3.192	0.078	3	0.489	0.691	3	0.869	0.461
**MUAC, cm**	81	**1**	1.013	0.318	3	0.643	0.591	3	0.474	0.702
**CRP, µmol/mL**	57	**1**	1.961	0.166	3	0.334	0.802	3	1.065	0.369
**LPS, EU/mL**	81	**1**	0.004	0.953	3	0.489	0.692	3	1.11	0.353
**sCD14, µmol/mL**	**83**	**1**	6.525	0.013	3	1.686	0.177	3	1.662	0.182
**Crypt Depth, µm**	**48**	**1**	15.632	0.001	3	0.392	0.76	3	1.173	0.331
**Villus Height, µm**	**48**	**1**	2.475	0.123	3	2.621	0.063	3	1.374	0.263
**Villus Width, µm**	**48**	**1**	0.345	0.561	3	0.959	0.421	3	1.23	0.310

Supplementary Table 2: Faiths PD Richness association with sample characteristics at baseline

	Duodenal Richness			Stool Richness		
**Term**	**Estimate**	**95% CI**	**p**	**Estimate**	**95% CI**	**p**
**Age (years)**	0.003	(-0.024, 0.029)	0.875	-0.031	(-0.075, 0.015)	0.189
**BMI**	-0.005	(-0.063, 0.053)	0.875	-0.01	(-0.114, 0.095)	0.864
**HIV Status: Positive**	0.011	(-0.003, 0.023)	0.142	-1.376	(-2.568, -0.182)	**0.027**
**Sex: Male**	0.003	(-0.006, 0.012)	0.518	-1.085	(-2.251, 0.081)	0.073
**CRP, µmol/mL**	0	(0, 0)	0.102	0	(0, 0)	0.09

**LPS, EU/mL**	0	(-0.003, 0.003)	0.928	0	(-0.006, 0.005)	0.954
**sCD14, µmol/mL**	0	(0, 0)	0.82	0	(-0.001, 0)	0.213
**Crypt Depth, µm**	0.013	(-0.733, 0.757)	0.975	0.003	(-0.015, 0.02)	0.795
**Villus Height, µm**	0.007	(-0.001, 0.014)	0.113	0.004	(-0.008, 0.014)	0.599
**Villus Width, µm**	0.006	(0, 0.011)	0.075	-0.003	(-0.012, 0.007)	0.614

Note: The estimate here represented the change in alpha diversity for every 1-unit shift in the sample characteristics. For example, duodenal richness increased non-significantly by 0.003 units for every unit increase in age. For categorical variables such as HIV status, this would be the unit difference in richness in that group compared to the reference. Therefore, stool richness in HIV positive individuals decreased by 1.38 units compared to HIV negative individuals.

Supplementary Table 3: PERMANOVA results of sample distances with sample characteristics

	**Duodenal Beta Diversity**			**Stool Beta Diversity**		
Term	**R2**	***F*-statistic**	**p**	**R2**	***F*-statistic**	**p**
**Age (years)**	0.052	3.606	**0.006**	0.019	1.421	0.166
**BMI**	0.028	1.779	0.093	0.023	1.624	0.103
**HIV Status: Positive**	0.025	1.663	0.111	0.03	2.311	**0.024**
**MUAC**						
**Sex: Male**	0.017	1.086	0.333	0.018	1.356	0.182
**CRP, µmol/mL**	0.016	1.098	0.336	0.017	1.302	0.217
**LPS, EU/mL**	0.014	0.788	0.565	0.007	0.460	0.912
**sCD14, µmol/mL**	0.038	2.607	**0.025**	0.025	1.953	0.058
**Crypt Depth, µm**	0.024	1.154	0.281	0.014	0.790	0.569
**Villus Height, µm**	0.018	0.853	0.542	0.01	0.532	0.84
**Villus Width, µm**	0.007	0.313	0.987	0.007	0.386	0.963

Note: The R^2 values here represents the percent of variation in the community that is explained by that sample characteristic. For example, Age has an R^2 value of .052 against duodenal diversity, therefore age accounts for 5.2% of the microbial variation seen in duodenal samples.

The *F*-statistic can be interpreted as a measure of between group variation. A higher *F*-statistic means there is greater variation between groups than within.

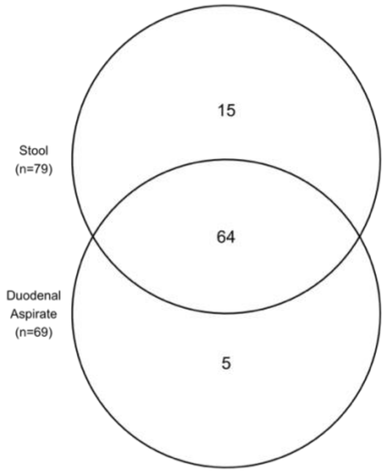

Supplementary Fig 1: A Venn diagram summarising of all samples used in the analysis for this paper.

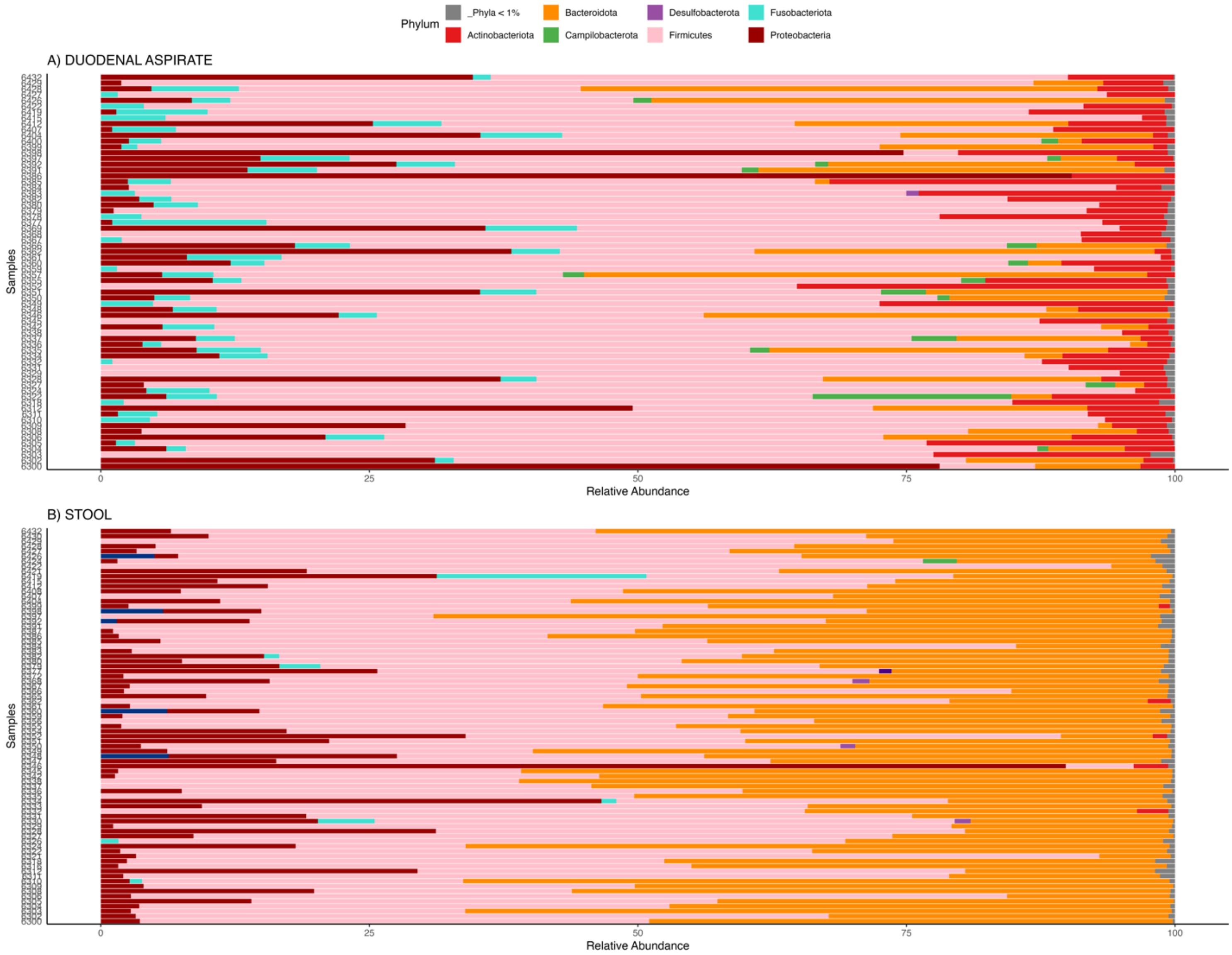

Supplementary Fig 2: Relative Abundance of gut phyla. A) in duodenal sample and b) stool samples.

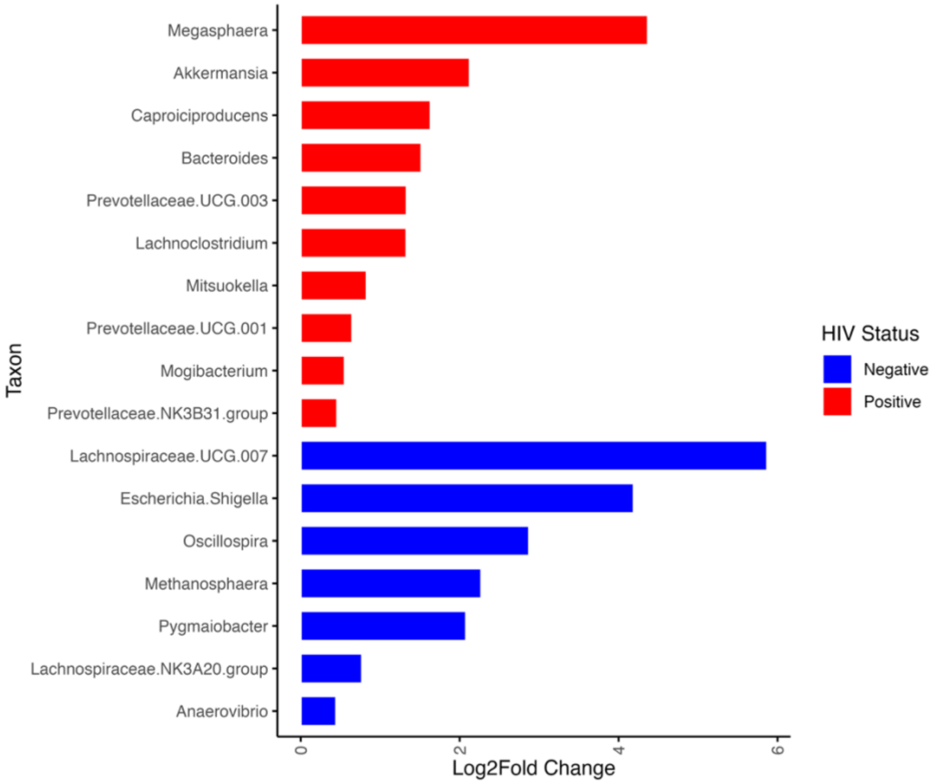

Supp Fig 3: Genera in stool with significant differential abundant by HIV status.

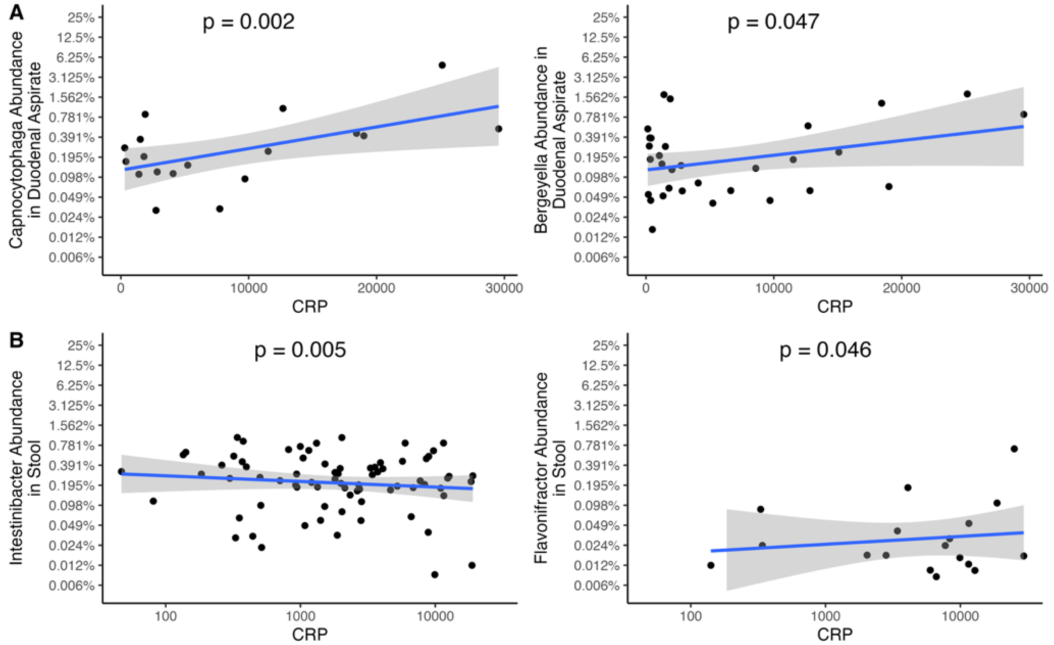

Supplementary Fig 4: Significant associations modelling the relationship between C-Reactive Protein (CRP) concentration in plasma and relative abundance of microbes. A) *Capnocytophaga* and *Bergeyella* abundance in duodenal aspirate and B) *Intestinibacter* and *Flavonifractor* abundance and in stool.

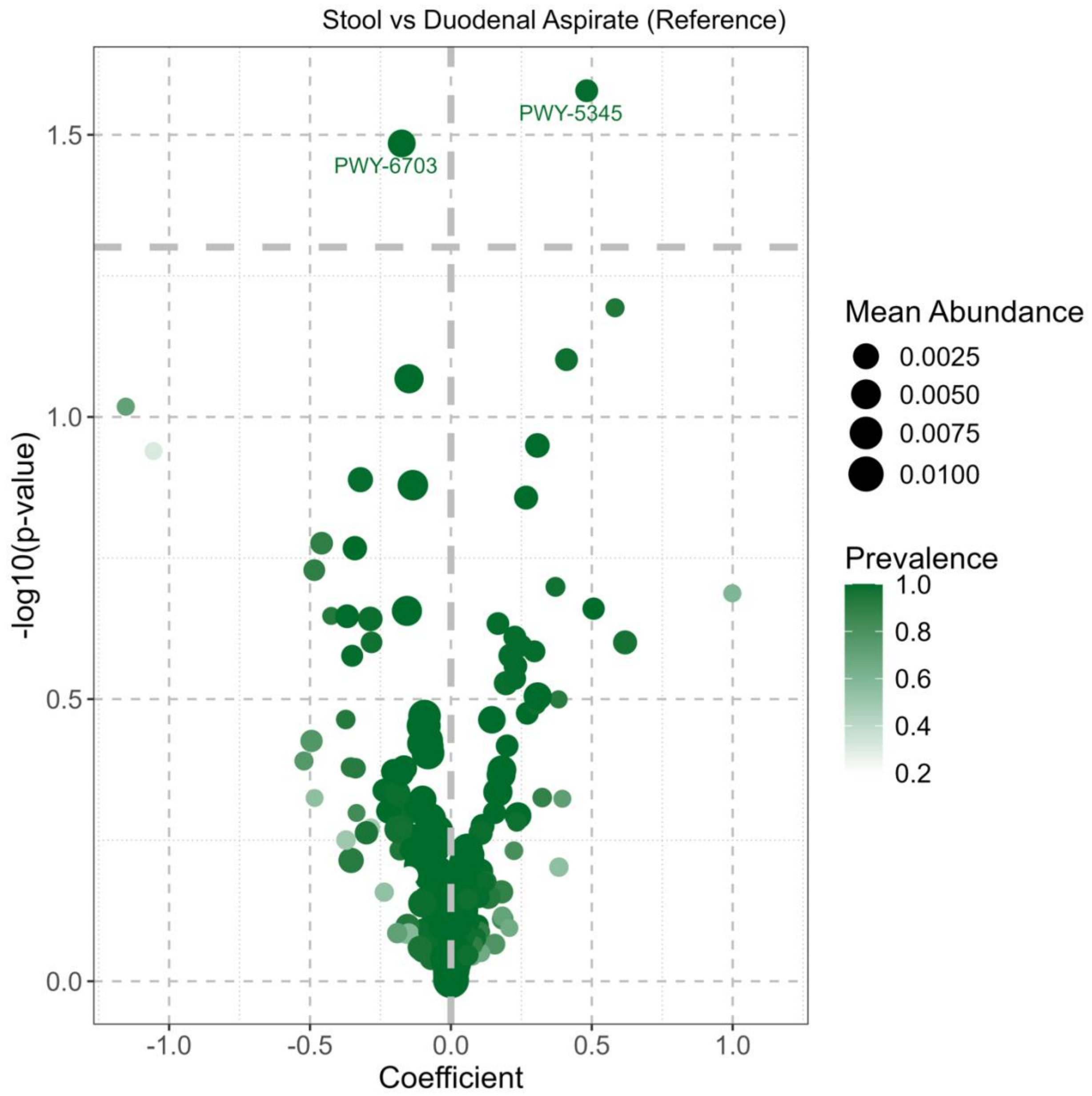

Supplementary Fig 5: Pathway associations with sample type at baseline. Metacyc IDs of significant pathways are labelled. The y-axis represents log (FDR corrected p-values). All points below the horizontal line (p=0.05) were not different between duodenal and fecal samples.

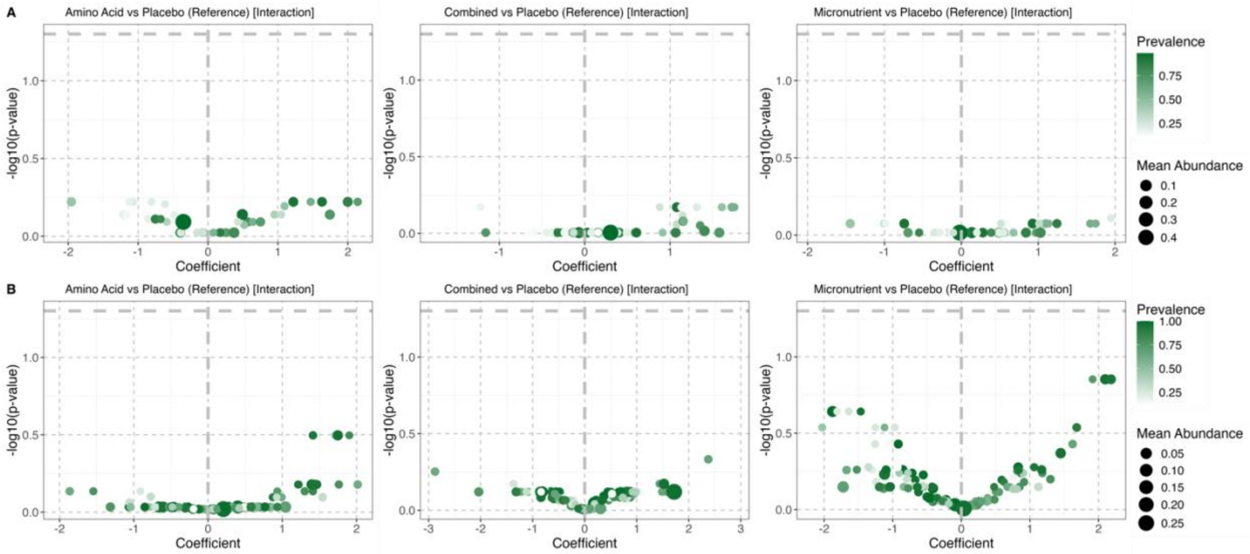

Supplementary Fig 6: Microbial changes in each nutritional arm compared to the placebo. A) in duodenal aspirate samples and B) in stool samples. The y-axis represents log (FDR corrected p-values). All points below the horizontal line (p=0.05) were not different between intervention arms and placebo

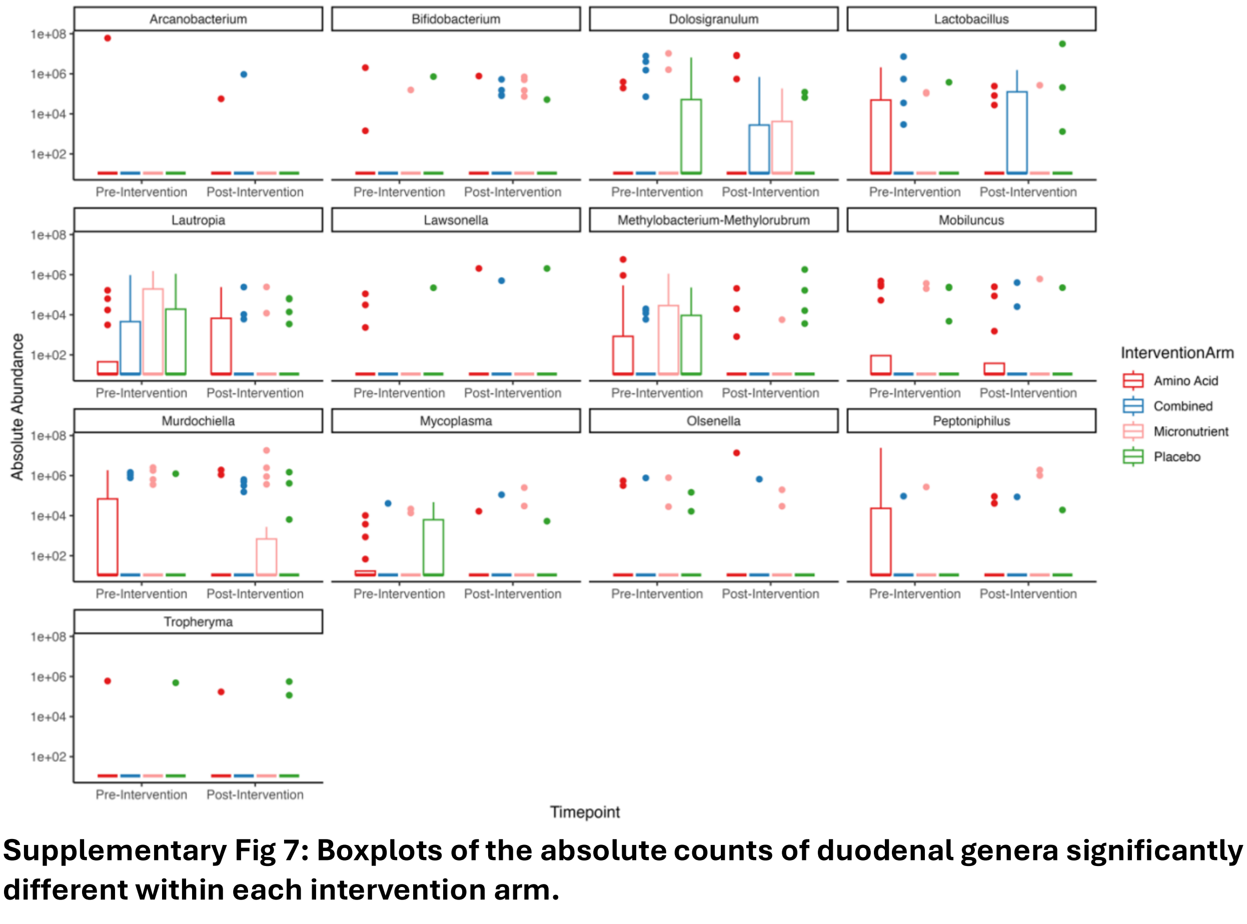

Supplementary Fig 7: Boxplots of the absolute counts of duodenal genera significantly different within each intervention arm.

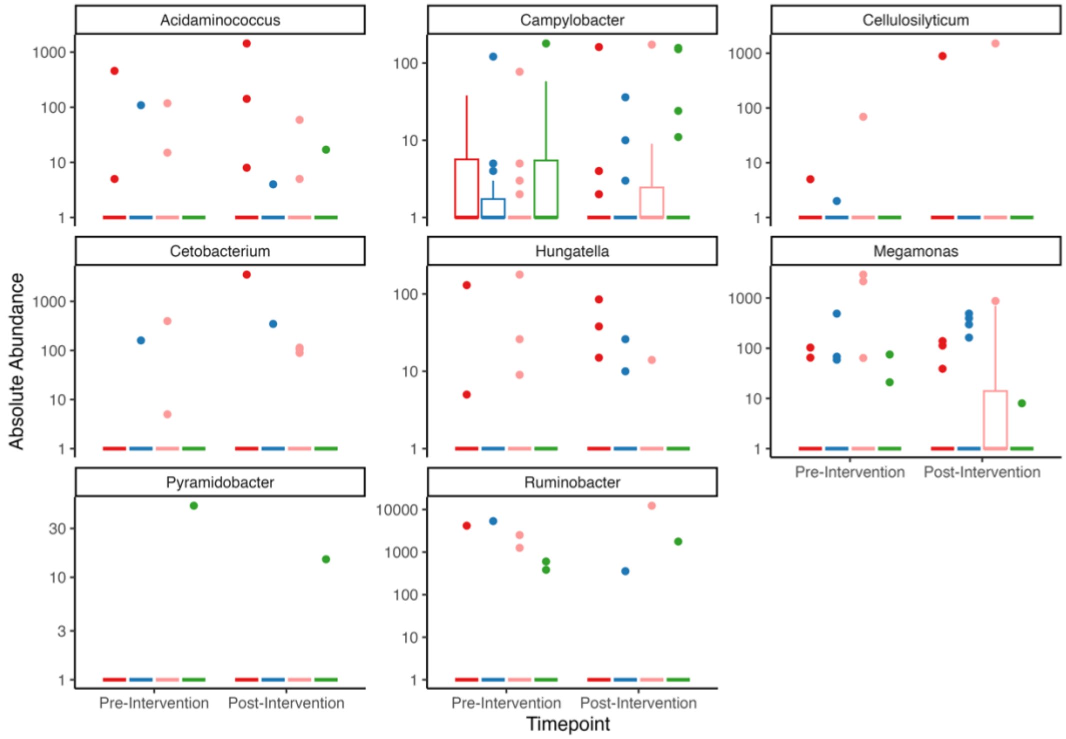

Supplementary Fig 8: Boxplots of the absolute counts of faecal genera significantly different within each intervention arm.

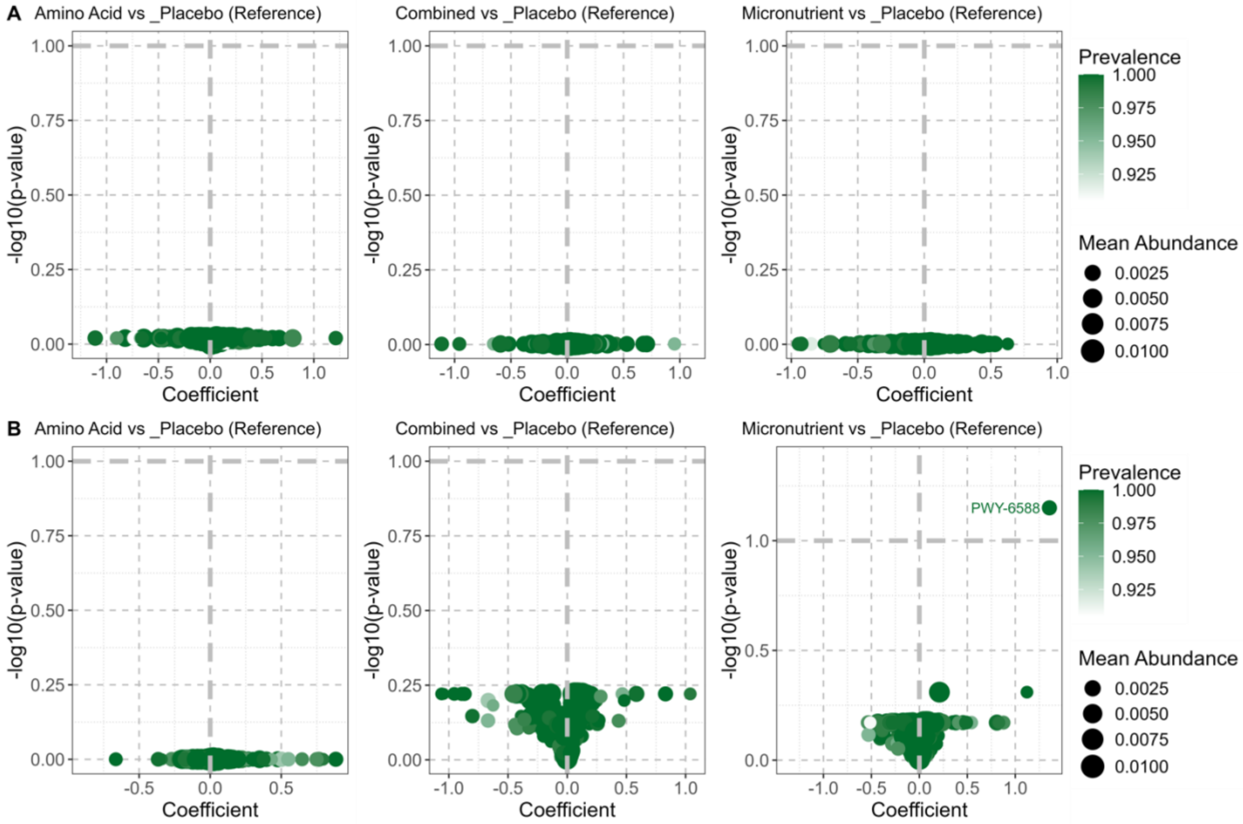

Supplementary Fig 9: Volcano plots of pathways differentially abundant in each nutritional arm compared to placebo. A) in duodenal aspirate samples and B) in faecal samples. The y-axis represents log (FDR corrected p-values). The horizontal dash line represents p=0.1 though p=0.05 was the cutoff for significance in the analysis.
